# Classification of idiopathic interstitial pneumonias

**DOI:** 10.1590/0100-3984.2018.51.5e1

**Published:** 2018

**Authors:** Alexandre Dias Mançano

**Affiliations:** 1 PhD, MD, Radiologist at the RA Radiologia, Brasília, DF, Brazil. E-mail: alex.manzano1@gmail.com.

Idiopathic interstitial pneumonias (IIPs) constitute a heterogeneous group of acute or chronic pulmonary diseases of unknown etiology, caused by lesion of the pulmonary parenchyma, resulting in varying degrees of inflammation and fibrosis^(1)^.

In 2002, the American Thoracic Society/European Respiratory Society (ATS/ERS) proposed the inclusion of seven entities in the IIP category—acute interstitial pneumonia, usual interstitial pneumonia (UIP), nonspecific interstitial pneumonia, desquamative interstitial pneumonia, respiratory bronchiolitis-associated interstitial lung disease, cryptogenic organizing pneumonia, and lymphoid interstitial pneumonia—defining clinical, radiological, and pathological criteria in the diagnosis of these diseases, as well as highlighting the importance of the multidisciplinary approach^(1)^. Since then, various studies have added to the body of knowledge about IIPs^(2-8)^.

In 2013, the ATS/ERS^(3)^ proposed some important changes in relation to the original (2002) IIP classification. Among the innovations are the subdivision of the IIPs into four main groups (chronic fibrosing, smoking-related, acute/subacute, and rare) and the addition of a new disease (in the rare subdivision): idiopathic pleuroparenchymal fibroelastosis. In addition to creating those subdivisions, the update included other important modifications in relation to the previous consensus. Nonspecific interstitial pneumonia came to be recognized as a distinct entity, with a variable, heterogeneous clinical course. Respiratory bronchiolitis-associated interstitial lung disease could now be diagnosed without the need for biopsy. Acute exacerbations of IIPs were more well defined. It was recognized that idiopathic pulmonary fibrosis (IPF) can have a heterogeneous clinical course. Another classification based on the clinical behavior of the disease was proposed for IIPs that present a mixed pattern of pulmonary injury or that present a heterogeneous clinical course. Finally, the update introduced a group of extremely rare diseases, such as idiopathic pleuroparenchymal fibroelastosis, and diseases with rare histological patterns.

The diagnosis of IIPs is often challenging^(2,4)^. Many pulmonary diseases of known etiology, especially those related to connective tissue diseases and hypersensitivity pneumonia, can show similar tomographic patterns, the clinical, radiological, and pathological correlations being fundamental for making the definitive diagnosis. In this context, the radiologist plays a fundamental role in this triad of the multidisciplinary diagnosis and follow-up of these diseases, and radiologists should therefore be able to recognize the various tomographic patterns related to IIPs^(2-5)^.

In an article published in this issue of **Radiologia Brasileira**—Idiopathic interstitial pneumonias: review of the latest American Thoracic Society/European Respiratory Society classification—Oliveira et al.^(9)^ revisit the topic in a didactic way, addressing the clinical, radiological, and pathological aspects of IIPs, as well as highlighting the main differential diagnoses. The authors provided clinical and pathological descriptions of the four main groups established in the 2013 ATS/ERS consensus^(3)^, illustrating the main tomographic patterns of IIPs by describing histopathologically confirmed cases. The Oliveira et al.^(9)^ article should be required reading for thoracic and general radiologists, because the recognition of certain tomographic patterns, correlated with the clinical profile, can be sufficient for making the definitive diagnosis, without the need for a biopsy.

In 2011, the ATS/ERS, in collaboration with the Japanese Respiratory Society and the Latin American Thoracic Association, published a new consensus on the diagnosis and management of IPF^(7)^. In that consensus, the tomographic criteria were established for the diagnosis of the UIP morphological pattern on high-resolution computed tomography (HRCT), based on the presence of four characteristics: subpleural, basal distribution; reticular opacities; honeycombing, with or without traction bronchiectasis; and the absence of morphological findings inconsistent with the UIP pattern, such as extensive ground-glass attenuation. In this context, patients could be diagnosed with IPF, according to clinical characteristics associated with the UIP pattern on HRCT, without the need for biopsy, greatly increasing the degree of responsibility that radiologist have for recognizing the UIP pattern on HRCT.

In 2017, the Fleischner Society published a white paper proposing diagnostic criteria for IPF^(8)^. In that paper, HRCT continues to play a key role in the diagnostic algorithm, and four tomographic patterns were proposed: typical UIP; probable UIP; undetermined UIP; and HRCT findings that are more consistent with a diagnosis other than IPF. The authors proposed that in the presence of the first two HRCT patterns, biopsy would no longer be necessary for the diagnosis of IPF, given the appropriate clinical context.

The development of a new generation of antifibrotic agents has increased the survival of patients with IPF. In addition, radiologists have come to play a greater role in multidisciplinary interstitial lung disease groups, because the correct reading of CT scans is crucial for making an accurate diagnosis.
